# The low-density lipoprotein receptor-related protein 1 and amyloid-β clearance in Alzheimer’s disease

**DOI:** 10.3389/fnagi.2014.00093

**Published:** 2014-05-20

**Authors:** Takahisa Kanekiyo, Guojun Bu

**Affiliations:** Department of Neuroscience, Mayo Clinic, JacksonvilleFL, USA

**Keywords:** Alzheimer’s disease, apolipoprotein E, amyloid-β, clearance, endocytosis, degradation, LRP1, signaling pathway

## Abstract

Accumulation and aggregation of amyloid-β (Aβ) peptides in the brain trigger the development of progressive neurodegeneration and dementia associated with Alzheimer’s disease (AD). Perturbation in Aβ clearance, rather than Aβ production, is likely the cause of sporadic, late-onset AD, which accounts for the majority of AD cases. Since cellular uptake and subsequent degradation constitute a major Aβ clearance pathway, the receptor-mediated endocytosis of Aβ has been intensely investigated. Among Aβ receptors, the low-density lipoprotein receptor-related protein 1 (LRP1) is one of the most studied receptors. LRP1 is a large endocytic receptor for more than 40 ligands, including apolipoprotein E, α2-macroglobulin and Aβ. Emerging *in vitro* and *in vivo* evidence demonstrates that LRP1 is critically involved in brain Aβ clearance. LRP1 is highly expressed in a variety of cell types in the brain including neurons, vascular cells and glial cells, where LRP1 functions to maintain brain homeostasis and control Aβ metabolism. LRP1-mediated endocytosis regulates cellular Aβ uptake by binding to Aβ either directly or indirectly through its co-receptors or ligands. Furthermore, LRP1 regulates several signaling pathways, which also likely influences Aβ endocytic pathways. In this review, we discuss how LRP1 regulates the brain Aβ clearance and how this unique endocytic receptor participates in AD pathogenesis. Understanding of the mechanisms underlying LRP1-mediated Aβ clearance should enable the rational design of novel diagnostic and therapeutic strategies for AD.

## INTRODUCTION

The low-density lipoprotein receptor (LDLR) family consists of structurally related single transmembrane receptors, including LDLR, LDLR-related protein 1 (LRP1), LRP1B, megalin/LRP2, very-LDLR (VLDLR), apolipoprotein E receptor 2 (ApoER2)/LRP8, sortilin-related receptor (SorLA/LR11), LRP5, and LRP6 (Herz and Bock, 2002; Jaeger and Pietrzik, 2008; Holtzman et al., 2012). These cell surface receptors recognize extracellular ligands for subsequent signaling and/or trafficking to either degradation or recycling pathways (Bu, 2009; Holtzman et al., 2012). While the LDLR family members often recognize the same ligands, they regulate distinct physiological or pathophysiological pathways due to unique tissue expression patterns (Krieger and Herz, 1994). In particular, apolipoprotein E (apoE), which transports cholesterol, is a critical ligand for several receptors of the LDLR family (Herz and Bock, 2002; Bu, 2009). Since the *APOE* ∊4 allele increases the risk for late-onset Alzheimer’s disease (AD) compared with the *APOE* ∊2 and ∊3 alleles (Corder et al., 1993; Farrer et al., 1997), LDLR family has been vigorously studied as a target to explore the complex pathogenesis of AD.

Amyloid-β (Aβ) peptides cleaved from amyloid precursor protein (APP) are the key molecules involved in AD pathogenesis; deposition of Aβ in the brain as senile plaques and cerebral amyloid angiopathy (CAA) likely triggers a cascade of events leading to disease onset (Hardy and Selkoe, 2002; Blennow et al., 2006). Recent evidence has also shown that soluble Aβ oligomers injure synapses resulting in cognitive impairment prior to Aβ deposition (Mucke and Selkoe, 2012). While familial AD, which accounts for ~1% of AD cases, is likely caused by genetic mutations in *APP*, *PSEN1*, and *PSEN2* leading to enhanced Aβ production (Thies and Bleiler, 2013), a positive correlation between Aβ levels and APP processing is not evident in sporadic late-onset AD (Shinohara et al., 2014), which represents the bulk of all AD cases. In fact, the disturbance of Aβ clearance machinery appears to be a leading cause of Aβ accumulation in the brain (Mawuenyega et al., 2010). Thus, the dysregulation of Aβ clearance pathways may be a central disease event in the majority of AD cases. Improved understanding of such pathways should help to both understand the complex pathogenesis of AD and allow for rationale design for AD therapy.

Among the LDLR family members, LRP1 is the most studied receptor due to its involvement in multiple pathways in AD pathogenesis (Zlokovic et al., 2010; Spuch et al., 2012). LDLR also mediates Aβ metabolism (Kim et al., 2009; Basak et al., 2012) and SorLA/LR11, which controls APP trafficking/processing (Andersen et al., 2005), is genetically associated with AD (Rogaeva et al., 2007). LRP1 is a large multi-functional receptor that regulates the endocytosis of diverse ligands and transduces several cell signaling pathways by coupling with other cell surface receptors. LRP1 is detected in most tissues and is highly expressed in liver, brain and lung. In the central nervous system, LRP1 is abundantly expressed in neurons, glial cells and vascular cells, and plays a critical role in maintaining brain homeostasis (Herz and Strickland, 2001; Lillis et al., 2008). In this review, we discuss how LRP1 regulates AD pathogenic pathways in different cell types with particular focus on Aβ clearance pathways.

## LRP1 AND ALZHEIMER’S DISEASE

### LRP1: STRUCTURAL AND FUNCTIONAL FEATURES

Low-density lipoprotein receptor-related protein 1 was initially identified in liver cells as LDLR homology (Herz et al., 1988). It is composed of two subunits including an 85-kDa C-terminal transmembrane domain and a 515-kDa N-terminal extracellular domain. LRP1 is synthesized as a glycosylated precursor protein and then cleaved into two subunits in the Golgi complex. After proteolytic processing, the extracellular domain of LRP1 is non-covalently connected to the transmembrane domain as it matures to the cell surface (Kowal et al., 1989). The extracellular domain of LRP1 contains four ligand-binding domains I-IV with 2, 8, 10, and 11 cysteine-rich complement-type repeats, respectively (Neels et al., 1999). These motifs contain a net negative charge, which allows the bindings of a variety of positively charged ligands (Spuch et al., 2012). The domains II and IV of LRP1 are the major binding regions (Obermoeller-McCormick et al., 2001). The cytoplasmic tail of LRP1 contains two copies of NPXY motifs, which commonly present in most members of the LDLR family and serve as the endocytosis signal for the LDLR (Krieger and Herz, 1994). In addition to the two NPXY motifs, the LRP1 cytoplasmic tail has a YXXL motif, which along with two di-leucine motifs serve as the dominant endocytosis signals for its rapid endocytosis (Li et al., 2000). When the endocytosis rates of several LDLR family members were compared using *in vitro* cellular models, the LRP1 tail showed faster endocytosis with t_1/2_ of ~0.5 min compared with those of the LDLR tail (*t*_1/2_ = 4.8 min) and megalin/LRP2 tail (*t*_1/2_ = 3.1 min), whereas VLDLR and ApoER2 exhibit relatively slower endocytosis rates (*t*_1/2_ = ~8 min; Li et al., 2001). Thus, the main function of LRP1 is to capture its ligands through extracellular ligand-binding domains, rapidly internalize them through its unique cytoplasmic tail and deliver them to the endosomal/lysosomal compartments. After dissociation of ligands in the early endosome, LRP1 is known to efficiently recycle back to the cell surface by coupling with sorting nexin 17 (van Kerkhof et al., 2005).

Low-density lipoprotein receptor-related protein 1 also regulates signaling pathways in response to extracellular ligands by several mechanisms (Gonias and Campana, 2014). Binding of tissue-type plasminogen activator (tPA) or α2-macroglobulin (α2M) to LRP1 induces Src family kinase (SFK) activation and SFK-dependent Trk receptor transactivation in neuronal cells (Shi et al., 2009). LRP1 also controls cell signaling by mediating the endocytosis of preformed receptor-ligand complexes into endosomes as a co-receptor. For example, LRP1 couples with the platelet-derived growth factor (PDGF) receptor and traffics into endosomes, where the phosphorylation of the PDGF receptor is induced in the presence of PDGF (Muratoglu et al., 2010). In addition, LRP1 modifies the distribution of several membrane proteins between the cell surface and intracellular pools, which impacts their signaling strength (Gonias et al., 2004). In some cases, LRP1 deletion appears to increase total, or cell surface, levels of PDGF receptor (Boucher et al., 2003) and urokinase plasminogen activator (uPA) receptor (Weaver et al., 1997), and activate their downstream signaling pathways. In neurons, LRP1 interacts with the *N*-methyl-D-aspartate (NMDA) receptor through PSD-95, and regulates its trafficking to recycling compartments or degradation pathway. When LRP1 is deleted in neurons, degradation of the NMDA receptor is accelerated, resulting in decreased NMDA receptor levels and disturbed signaling pathways (May et al., 2004; Maier et al., 2013). Furthermore, the C-terminal intracellular domain of LRP1 (LRP1-ICD), cleaved from the transmembrane domain by γ-secretase, likely regulates the transcription of target genes (Spuch et al., 2012). In fact, it has been demonstrated that LRP1-ICD suppresses LPS-induced inflammatory responses by binding to the interferon-γ promoter (Zurhove et al., 2008).

Taken together, LRP1 serves as a multifunctional receptor that controls the endocytosis of a variety of ligands, influences signaling pathways by coupling with other cell surface receptors or proteins, and directly regulates gene expression through its intracellular domain.

### LRP1 IN AD PATHOGENESIS

Low-density lipoprotein receptor-related protein 1 ligands, specifically apoE, α2M, tPA, uPA, plasminogen activator inhibitor-1, lipoprotein lipase, and lactoferrin co-deposit with Aβ in senile plaques in AD brains (Namba et al., 1991; Rebeck et al., 1995). In fact, the soluble form of LRP1 is also likely a prominent component of senile plaques and has been found to co-localize exclusively with these ligands (Rebeck et al., 1995). Although it is not clear whether LRP1 and its ligands are independently associated with senile plaques, these observations suggest that they do interact with Aβ in AD brains. Immunohistochemical analysis has revealed that LRP1 is up-regulated in neurons and in GFAP-positive activated astrocytes, in particular in astrocytic processes surrounding senile plaques in AD (Arelin et al., 2002). Consistent with this finding, LRP1 mRNA levels are shown to be increased in temporal neocortex in AD patients (Matsui et al., 2007). Since the mRNA levels of both LRP1 and GFAP are up-regulated in AD brains with a positive correlation (Matsui et al., 2007), LRP1 expression is likely enhanced in activated astrocytes. On the other hand, LRP1 levels have been shown to be decreased in midfrontal cortex in AD cases (*n* = 39) as compared to age-matched controls (*n* = 39; Kang et al., 2000). Interestingly, higher LRP1 levels significantly correlate with later ages at onset of AD, while age and LRP1 expression in normal individuals appears inversely correlated (Kang et al., 2000). In addition, when the regional correlations between LRP1 and synaptic markers (synaptophysin and PSD95) or GFAP were assessed, a moderate-to-strong positive regional correlation was observed between LRP1 and postsynaptic marker PSD95, but not presynaptic marker synaptophysin and GFAP in the brains from non-demented individuals (Shinohara et al., 2013). Thus, LRP1 expression may be either up-regulated in glial cells due to neuroinflammation or suppressed in neurons due to postsynaptic damages in AD. Further studies are needed to clarify the temporal and spatial regulation of LRP1 expression in AD brains.

Several lines of evidence support a role of LRP1 in regulating APP endocytosis. LRP1 can bind to the Kunitz-type protease inhibitor (KPI) domain of APP and mediate its degradation (Kounnas et al., 1995). The association of APP with LRP1 leads to increased trafficking of APP through the endosomal compartments, resulting in accelerated Aβ production and APP processing (Ulery et al., 2000; Cam et al., 2005). Consistent with these results, the regional correlation between LRP1, APP, and Aβ showed positive correlations in non-demented individuals (Shinohara et al., 2013). Therefore, LRP1 may function to increase Aβ levels from the perspective of APP processing and Aβ production. On the other hand, LRP1 plays a critical role as an endocytic receptor to eliminate Aβ from the brain. In fact, after intracerebral microinjections of [^125^I]-Aβ40 in young mice, Aβ was rapidly removed from the brain (*t*_1/2_ < 25 min). Aβ40 clearance was significantly inhibited by LRP1 antagonist, RAP, or antibodies against LRP1 (Shibata et al., 2000). Thus, the ability of LRP1 to regulate both Aβ production and clearance suggests a critical role of this receptor in AD pathogenesis. In Aβ-independent pathways, conditional deletion of the *Lrp1* gene in forebrain neurons in mice leads to age-dependent dendritic spine degeneration, synapse loss, neuroinflammation, memory loss, and neurodegeneration (Liu et al., 2010), which are all common features of AD.

### RELATIONSHIP AMONG LRP1, APOE, AND Aβ IN AD

Low-density lipoprotein receptor-related protein 1 is a major apoE metabolic receptor in the brain (Zerbinatti et al., 2006; Liu et al., 2007). As *APOE4* dramatically increases AD risk and accelerates disease onset compared with *APOE2* and *APOE3* (Bu, 2009; Liu et al., 2013), understanding how apoE is involved in AD pathogenesis has been both an opportunity and a challenge. Given that apoE4 is related to increased Aβ aggregation and deposition in the brain (Bu, 2009; Liu et al., 2013), apoE-Aβ interaction has been actively studied to understand the specific roles of apoE isoforms. While the main function of apoE is to transfer lipid from cell to cell through cell surface LRP1 and other apoE receptors, apoE can also bind to Aβ through a region overlapping with its receptor-binding site (Winkler et al., 1999) or lipid-binding site (Strittmatter et al., 1993) in an isoform-dependent manner (Kanekiyo et al., 2014). Although growing evidence supports apoE-Aβ binding, a recent report showed that only a small portion of soluble, cell-derived Aβ interacted with astrocyte-secreted or artificially reconstituted apoE particles in solution (Verghese et al., 2013). Thus, the effects of apoE on Aβ cellular uptake are likely complex. While recombinant apoE accelerates neuronal Aβ uptake in an isoform-dependent manner (apoE3 > apoE4; Li et al., 2012), apoE particles inhibited the cellular uptake of soluble Aβ in astrocytes (Verghese et al., 2013). Of note, the suppressive effect of apoE particles on Aβ uptake was not detected in LRP1-deficient cells. The LRP1-blocking antibody also significantly decreased the effect of apoE on Aβ uptake in astrocytes (Verghese et al., 2013). Thus, apoE may either facilitate or inhibit LRP1-dependent or independent Aβ endocytosis depending on its concentration, Aβ aggregation state, apoE isoform, apoE lipidation and the expression pattern of the receptors on the cell surface (Kanekiyo et al., 2014).

The activated form of α2M (α2M*) is also a well validated LRP1 ligand (Strickland et al., 1990). While α2M* associates with Aβ and prevents fibril formation (Hughes et al., 1998), α2M* enhances the clearance of soluble Aβ via LRP1 in neurons (Narita et al., 1997; Qiu et al., 1999). RAP, which is an LRP1 chaperone and antagonist, can also interact with Aβ and facilitate its cellular uptake through heparan sulphate proteoglycan (HSPG), rather than LRP1 (Kanekiyo and Bu, 2009). Thus, it is interesting to note that several major LRP1 ligands can bind to Aβ, suggesting the existence of common mechanisms by which Aβ and other ligand interact with LRP1 and/or HSPG. Further biochemical and structural studies are needed to determine the binding properties among Aβ, LRP1 and its ligands, which may provide insights as to the differential effects of LRP1 ligands on cellular Aβ metabolism.

## LRP1 AND BRAIN Aβ CLEARANCE

### LRP1-MEDIATED Aβ CLEARANCE IN BRAIN PARENCHYMA

**FIGURE 1:**
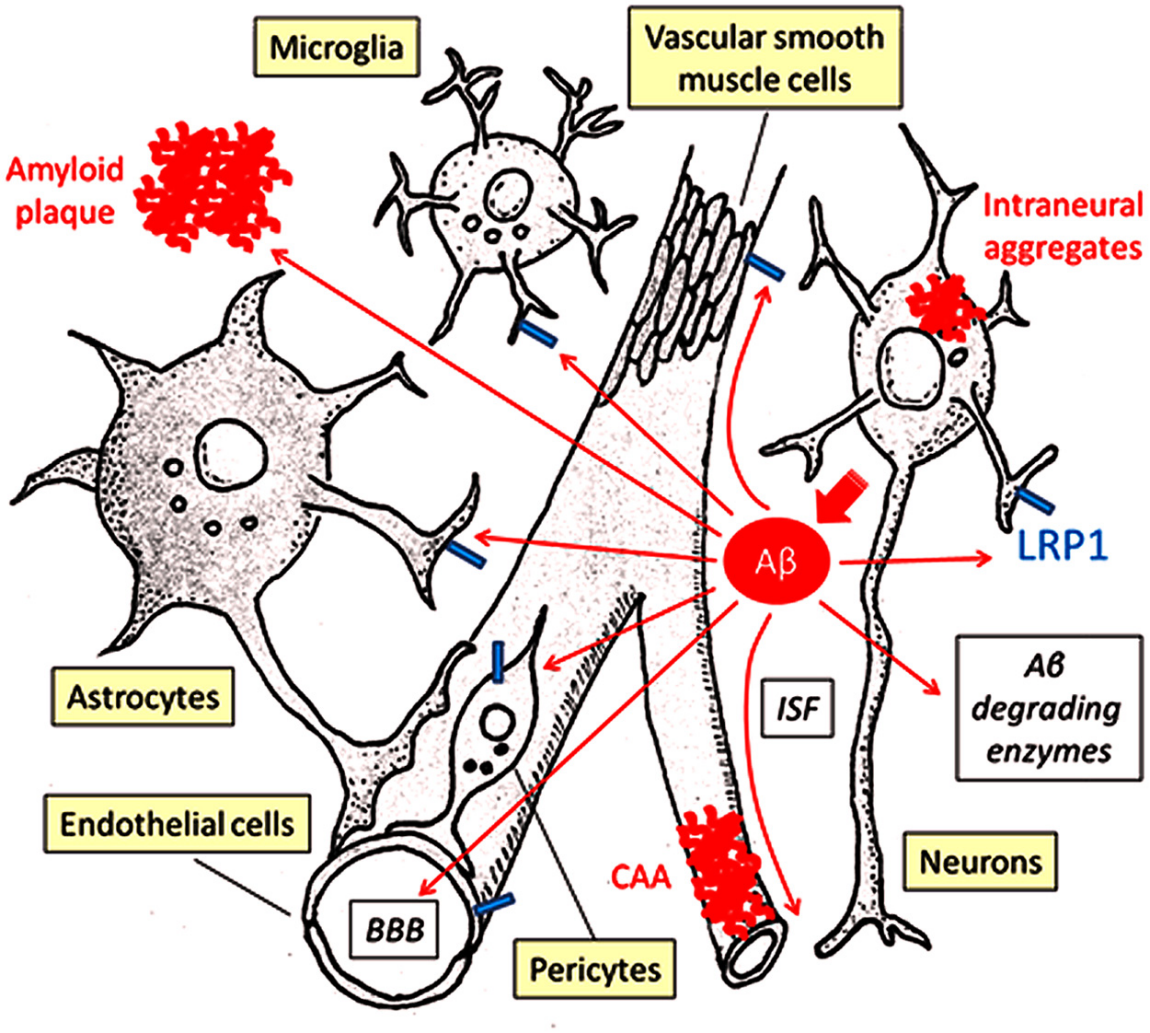
**LRP1-mediated Aβ clearance pathways**. Aβ is predominantly generated in neurons and secreted into ISF. Proteolytic degradation by endopeptidases (e.g., neprilysin, insulin-degrading enzyme) comprises a major Aβ clearance pathway. Cellular Aβ clearance also plays a critical role in eliminating Aβ from the brain, where LRP1 significantly regulates its endocytosis and subsequent lysosomal degradation. LRP1 is expressed in several different brain cell types, including neurons, astrocytes, microglia, endothelial cells, and vascular mural cells (vascular smooth muscle cells and pericytes). In brain parenchyma, neurons, astrocytes, and microglia can take up and degrade Aβ mainly in lysosomes. ISF is drained along the cerebrovasculature, where Aβ is degraded by vascular cells. A portion of Aβ may be transported out of the brain through the BBB. Disturbances of these pathways induce Aβ accumulation and deposition as amyloid plaques in brain parenchyma, perivascular regions as CAA and sometimes also inside neurons and intraneuronal Aβ.

Cellular Aβ clearance through lysosomal degradation in brain parenchyma cells (microglia, astrocytes, neurons) and in cerebrovascular system constitutes a major pathway (**Figure 1**), while Aβ is also efficiently degraded by a large set of proteases including neprilysin and insulin-degrading enzyme in extracellular space (**Figure 1**; Saido and Leissring, 2012). Neurons not only produce Aβ from APP proteolytic processing but also eliminate it through cellular uptake and lysosomal degradation (Li et al., 2012). If neuronal Aβ endocytosis is disturbed, the accumulation and aggregation of Aβ may lead to synaptic injury and eventual neuronal death. In neurons, LRP1 is expressed predominantly in the postsynaptic region (May et al., 2004) and the cell body (Bu et al., 1994), where LRP1 mediates Aβ uptake and subsequent degradation (**Figure 1**; Kanekiyo et al., 2011, 2013). When LRP1 is deleted in neurons in adult mice, the half-life of interstitial fluid (ISF) Aβ in cortex increases, resulting in exacerbated amyloid pathology (Kanekiyo et al., 2013). In addition, it is interesting to note that upon internalization from distal axons, Aβ can also be transported to neighboring neurons after secretion through exosomes (Song et al., 2014). Pharmacological inhibition of dynamin-mediated endocytosis leads to accumulation of Aβ on the cell surface and further prevents the transneuronal transmission of Aβ (Song et al., 2014). Although potential involvement of LRP1 in the Aβ transcytosis pathway is not clear, it is tempting to speculate that the cellular uptake of Aβ through LRP1-dependent pathway might be an important step. In this regard, it might be interesting to test the effect of neuronal LRP1 deletion on Aβ propagation pathway. While lysosome has a strong ability to degrade Aβ, any disturbances of this pathway or when the accumulation of Aβ exceeds its degradation capacity could lead to Aβ aggregation in the lysosomes (Hu et al., 2009; Li et al., 2012), thus accelerating intraneuronal Aβ aggregation and deposition (Eimer and Vassar, 2013). In fact, lysosomal enzymes, cathepsins B and D, seem to be reduced when Aβ accumulates in the lysosomes of amyloid model mice (Torres et al., 2012). In addition, if these lysosome-initiated Aβ aggregates are spread through neuronal connections, it may contribute to the propagation of Aβ aggregation as well as neuronal toxicity.

Cellular Aβ uptake by glial cells (i.e., astrocytes and microglia) is likely to represent alternative Aβ clearance pathways (**Figure 1**). When adult mouse astrocytes are co-cultured with brain sections from amyloid model mice containing Aβ deposition, Aβ levels in these sections are reduced (Wyss-Coray et al., 2003). Excessive astrocyte activation is a common pathological feature of AD (Verkhratsky et al., 2010); while activated astrocytes promote neurodegeneration (Verkhratsky et al., 2010), they might also have protective functions by facilitating Aβ clearance. Interestingly, exogenous adult astrocytes can efficiently eliminate Aβ in an apoE-dependent manner, perhaps in a manner that depends on LRP1 function (Koistinaho et al., 2004). Several *in vitro* experiments have also shown that LRP1 controls Aβ uptake in astrocytes and further mediates Aβ-induced astrocyte activation (LaDu et al., 2000). *In vivo* studies using astrocyte-specific *Lrp1* knockout mice should address the specific role of LRP1 in astrocyte-mediated Aβ clearance.

In microglia, soluble Aβ is likely internalized by fluid-phase macropinocytosis into lysosomes (Mandrekar et al., 2009). On the other hand, microglia takes up larger Aβ fibrils through phagocytosis via a multi-component cell surface receptor complex (Bamberger et al., 2003). Importantly, the uptake of Aβ-coated yeast particles in microglia was suppressed by the presence of LRP1 ligands including lactoferrin, α2M* or RAP (Laporte et al., 2004), indicating that LRP1 might regulate Aβ phagocytosis (**Figure 1**). LRP1 is also shown to mediate phagocytosis of apoptotic cells by binding to cell surface calreticulin in macrophages (Gardai et al., 2005), suggesting that LRP1 may play a role in eliminating apoptotic cells containing Aβ. In addition, ABCA7, another membrane protein that is implicated in AD risk, co-localizes with LRP1 on cell surface and enhances the phagocytosis of apoptotic cells through LRP1 in macrophages (Jehle et al., 2006). Whereas CR1 is a receptor for the complement fragments C3 and C4b (Crehan et al., 2012), LRP1 directly binds to C1q which triggers a complement activation cascade (Duus et al., 2010), affecting phagocytic function. Of note, several inflammation-related genes expressed in macrophage/microglia, including *TREM2*, *CD33*, *CR1*, and *ABCA7*, have been shown to be related to the risk of late-onset AD and are generating great interests in the AD research community (Harold et al., 2009; Lambert et al., 2009; Hollingworth et al., 2011; Naj et al., 2011; Guerreiro et al., 2013). Further studies may demonstrate cooperative roles among these GWAS-identified molecules and LRP1 for Aβ clearance in microglia. Interestingly, LRP1-deletion exacerbates inflammation by activating the NFκB pathway in peripheral macrophages (Gaultier et al., 2008). Thus, it is possible that LRP1 also regulates Aβ uptake in glial cells by controlling inflammatory responses and phagocytic machinery.

### LRP1-MEDIATED Aβ CLEARANCE IN CEREBROVASCULATURE

The cerebrovascular system, which is composed of endothelial cells, vascular mural cells (i.e., vascular smooth muscle cells and pericytes) and astrocytes, plays critical roles in maintaining brain homeostasis and perturbations of this system lead to neuronal loss and cognitive decline (Zlokovic, 2011). Interestingly, epidemiological studies have clearly shown that cerebrovascular disturbances, including diabetes mellitus, atherosclerosis, stroke, hypertension, transient ischemic attacks, microvessel pathology, and smoking, are risk factors for AD (de la Torre, 2002). Vascular smooth muscle cells, which are attached to endothelial cells and covered by astrocytes, are a major component of intracerebral arteries. At the brain capillary level, vascular endothelial cells and pericytes attached to the basement membrane form the blood-brain barrier (BBB) together with astrocyte end-foot processes (Zlokovic, 2011). Importantly, the cerebrovascular system is yet another major pathway that mediates brain Aβ clearance by either transporting Aβ out of the brain via BBB or degrading it in vascular mural cells, which include vascular smooth muscle cells in cerebral arteries and pericytes in the capillaries (**Figure 1**; Marques et al., 2013; Sagare et al., 2013c).

Low-density lipoprotein receptor-related protein 1 is highly expressed in cerebrovasculature. In vascular smooth muscle cells, serum response factor and myocardin suppress Aβ clearance by down-regulating LRP1 (Bell et al., 2009). Our group has also directly demonstrated that conditional deletion of LRP1 in vascular smooth muscle cells in amyloid model mice exacerbated Aβ deposition as amyloid plaques and CAA (**Figure 1**; Kanekiyo et al., 2012). While LRP1 mediates lysosomal Aβ degradation in vascular smooth muscle cells (Kanekiyo et al., 2012), LRP1 may be involved in controlling the cerebrovascular function as a signal transducing receptor through coupling with other receptors as described in previous sections. Since the cerebroarterial pulsations provide the driving force for drainage of ISF along the cerebrovasculature (Schley et al., 2006), deletion of LRP1 in vascular smooth muscle cells may cause Aβ accumulation by disturbing overall cerebroarterial functions as well as cellular Aβ clearance. In addition, pericyte loss caused by haploinsufficiency of *Pdgfrβ* gene suppresses Aβ clearance and accelerates Aβ deposition in amyloid model mice (Sagare et al., 2013b). Given that LRP1 mediates Aβ uptake and lysosomal degradation in cultured pericytes (Sagare et al., 2013b); LRP1 is likely a critical player in Aβ clearance within the cerebrovascular system (**Figure 1**).

Low-density lipoprotein receptor-related protein 1 is expressed in mouse brain capillaries and mediates Aβ binding, internalization, and clearance (**Figure 1**; Deane et al., 2004), although the LRP1-independent pathway is also shown (Ito et al., 2010). Vascular endothelial cells are major components of brain capillaries and BBB, which critically regulates the influx and efflux of components between cerebral ISF and blood flow (Zlokovic, 2011). Thus, BBB breakdown may lead to the disturbance of endothelial cell-mediated Aβ clearance across BBB. *In vitro* BBB model using primary cultures of mouse endothelial cells harboring mutated LRP1 endocytosis signal has shown that radiolabeled Aβ is transcytosed through LRP1 rather than degraded (Pflanzner et al., 2011). On the other hand, other groups using gene overexpressing or knockdown method have demonstrated that endothelial cell lines can internalize and degrade Aβ through LRP1 (Nazer et al., 2008; Yamada et al., 2008). Thus, further studies using endothelial cell-specific *Lrp1* knockout mice are needed to assess how much endothelial cell-internalized Aβ undergoes transcytosis through LRP1 at the BBB. LRP1 antisense treatment reduced Aβ clearance at the BBB, resulting in exacerbated brain Aβ accumulation and cognitive impairment (Jaeger et al., 2009). Interestingly, LRP1-mediated Aβ clearance at the BBB was reduced by apoE in an isoform-dependent manner (apoE4 > apoE3 > apoE2; Deane et al., 2008). Furthermore, apoE4 activates the cyclophilin A-MMP9 pathway through LRP1 in pericytes, leading to damages of BBB integrity (Bell et al., 2012). Thus, apoE isoforms differentially regulate Aβ clearance from the brain through several LRP1-regulated pathways in the cerebrovascular system.

In leptomeningeal arteries from AD patients with CAA, Western blot analysis revealed elevated Aβ levels and lower levels of LRP1 compared with age-matched, non-demented controls (Bell et al., 2009). Immunohistochemical analysis also showed that LRP1-positive vessels were reduced in patients with AD (Shibata et al., 2000) and cerebrovascular β-amyloidosis Dutch-type compared to controls (Deane et al., 2004), although there are also conflicting reports (Donahue et al., 2006; Wilhelmus et al., 2007). While Aβ exposure decreased LRP1 levels in endothelial cells in a dose-dependent manner (Deane et al., 2004), hypoxia or reactive oxygen species, conditions often detected in AD (Fanelli et al., 2013; Swerdlow et al., 2013), reduced LRP1 expression in vascular smooth muscle cells (Bell et al., 2009; Kanekiyo et al., 2012). In fact, LRP1 expression in brain microvasculature is known to decline in an age-dependent manner in mice (Silverberg et al., 2010). Therefore, aging or vascular damage may impair Aβ clearance by reducing LRP1 expression in the cerebrovascular system, which leads to eventual Aβ accumulation and aggregation as senile plaques and CAA. In addition, accumulation of copper in brain capillaries was associated with LRP1 reduction in mice. When mice were chronically treated with low levels of copper in their drinking water, copper disrupted brain Aβ clearance by decreasing LRP1 at BBB in a mouse model of AD (Gu et al., 2011; Singh et al., 2013). Thus, toxic chemical elements also appear to be involved in AD pathogenesis by influencing LRP1, although further studies are needed to clarify the pathways.

In addition, LRP1 likely mediates Aβ clearance at the blood-cerebrospinal fluid (CSF) barrier as well as BBB. After intracerebroventricular administration, radiolabeled Aβ was eliminated from the CSF with a half-life of 17.3 min, which was significantly suppressed in the presence of RAP or anti-LRP1 antibody (Fujiyoshi et al., 2011). These findings suggest that LRP1 is involved in the elimination of Aβ from CSF in epithelial cells at choroid plexus.

### SOLUBLE LRP1 AND Aβ CLEARANCE

The β-secretase BACE1 cleaves LRP1 on the cell surface, resulting in the release of the LRP1 extracellular domain termed soluble LRP1 (von Arnim et al., 2005). In addition, α-secretases ADAM10 and ADAM17 are also likely involved in LRP1 shedding. In MEF cells lacking ADAM10 and/or ADAM17, LRP1 shedding was significantly decreased, while overexpression of ADAM10 increased the release of soluble LRP1 (Liu et al., 2009). While the biological functions of soluble LRP1, which normally circulates in plasma (Quinn et al., 1997), are not fully understood, plasma soluble LRP1 level appears to be decreased in AD patients compared with control individuals (Sagare et al., 2007). Zlokovic’s group has shown that 70–90% of Aβ is bound to soluble LRP1 in plasma from cognitively normal individuals. Furthermore, the remaining soluble LRP1 in AD patients appears to be highly oxidized, which results in a lower binding affinity for Aβ (Sagare et al., 2007). Thus, soluble LRP1 may be a novel target as a plasma biomarker for AD, although the reliability of blood biomarkers has been questioned (Rosen et al., 2013). When recombinant soluble LRP1 domain IV (LRPIV) or LRPIV-D3674G mutant were administrated intraperitoneally into amyloid model mice for 3 months, brain Aβ levels were significantly decreased (Sagare et al., 2007, 2013a). Since these recombinant soluble LRP1 proteins do not cross BBB, they are predicted to eliminate brain Aβ through a peripheral Aβ sink mechanism. Decreasing peripheral Aβ levels likely drives Aβ transport across the BBB (Zlokovic, 2011), although there are conflicting reports on such a hypothesis (Walker et al., 2013; Henderson et al., 2014). Pharmacological approaches to increase circulating soluble LRP1 also appears to ameliorate amyloid pathology in amyloid model mice (Sehgal et al., 2012). In addition, it is interesting to note that LRPIV-D3674G increased cerebral blood flow responses to whisker stimulation in amyloid model mice (Sagare et al., 2013b). Further studies are again needed to investigate both the mechanism and the therapeutic value of soluble LRP1 in circulation.

## MECHANISMS OF LRP1-REGULATED CELLULAR Aβ UPTAKE

**FIGURE 2:**
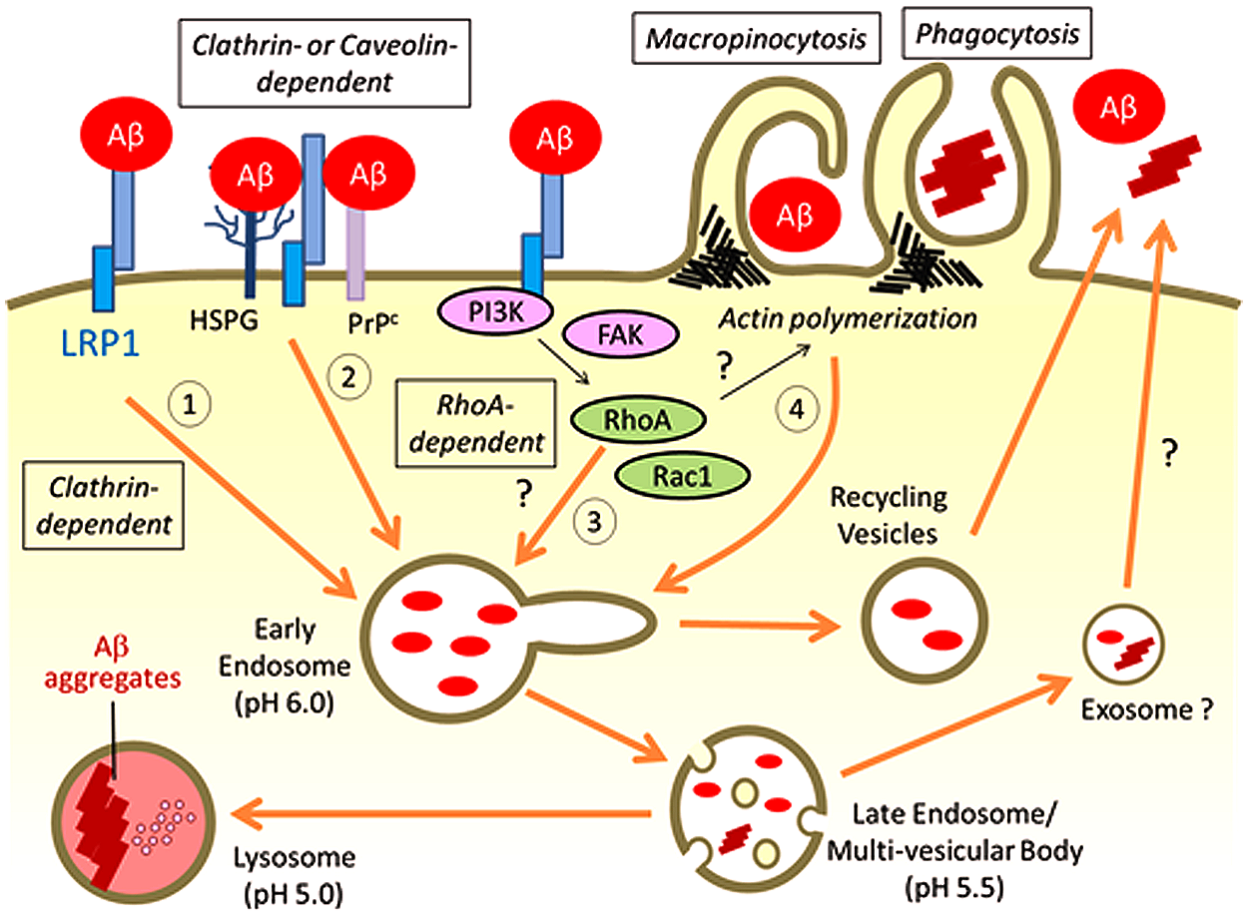
**Possible pathways for LRP1-mediated Aβ uptake**. LRP1 regulates Aβ internalization on the cell surface through several possible endocytic pathways: (1) Aβ binds to LRP1 directly for endocytosis through clathrin-dependent pathways; (2) Aβ binds to alternative cell surface receptors such as HSPG and PrP^c^ with LRP1 directly or indirectly regulating Aβ endocytosis in a clathrin- or caveolin-dependent manner; (3) LRP1 may affect RhoA-mediated endocytic pathway for cellular Aβ uptake by regulating signal transduction pathways; (4) LRP1 controls the cytoskeleton architectures by modifying PI3K and/or FAK pathways, which may influence macropinocytosis or phagocytosis of Aβ in specific cell types. The majority of endocytosed Aβ traffics to lysosomes for subsequent degradation, whereas a small amount of Aβ can be recycled. Under some conditions, Aβ is likely to be exocytosed from the late endosomes/multi-vesicular body, which may induce propagation of Aβ aggregates. When Aβ accumulation overwhelms the capacity of lysosomes for degradation, Aβ aggregation may be induced in lysosomes.

Internalized Aβ has been shown to predominantly traffic through the endosome/lysosome pathways for degradation (**Figure 2**; Basak et al., 2012; Lee et al., 2012; Li et al., 2012). Overexpression of small GTPases Rab5 and Rab7, which regulates vesicle fusion for early and late endosomes, respectively, facilities the trafficking of Aβ into lysosomes (Li et al., 2012). Blocking the late endocytic pathway by Rab7 knockdown induced the enlargement of early endosomes and amyloid fibril formation (Yuyama and Yanagisawa, 2009). In addition, a small portion of endocytosed Aβ likely traffics through the recycling vesicles (**Figure 2**) as a blockage of this pathway by a constitutively active Rab11 mutant significantly accelerated cellular Aβ accumulation in the recycling endosomes (Li et al., 2012).

Recent GWAS studies have also identified several endocytosis-related genes, including *BIN1, PICALM*, and *CD2AP*, as novel AD risk genes (Harold et al., 2009; Lambert et al., 2009; Naj et al., 2011). These genes are likely involved in clathrin-mediated endocytosis and vesicular trafficking to the lysosome (Guerreiro and Hardy, 2011). Thus, the altered endocytosis pathways likely contribute to modifying AD pathology, although it is unclear whether LRP1 function is related to these genes. LRP1 is a major clathrin-dependent endocytic receptor (Spuch et al., 2012). We have shown that the endocytic function of LRP1 is required for neuronal Aβ uptake (Fuentealba et al., 2010). Overexpression of a functional LRP1 minireceptor, mLRP4, increased Aβ uptake in neuronal cells, where the effect is reversed when LRP1 endocytic function was disturbed by either clathrin knockdown or by removal of its cytoplasmic tail (Fuentealba et al., 2010). However, the endocytosis rate of Aβ was slower than another LRP1 ligand RAP (Kanekiyo et al., 2011). These results suggest that LRP1 plays an important role in regulating Aβ endocytosis, although it is possible that other mechanisms are also involved in the process (**Figure 2**). In fact, it is controversial whether LRP1 directly binds to Aβ for its endocytosis. Surface plasmon resonance (SPR) analysis showed the high binding affinity of Aβ40 to immobilized recombinant LRP1 fragments of its ligand-binding domains II and IV with Kd values of 0.57 ± 0.12 and 1.24 ± 0.01 nM, respectively. In case of Aβ42, the binding affinity for LRP1 was reduced compared with Aβ40, where Kd values for LRP II and IV domains were 3.00 ± 0.11 and 10.10 ± 0.03 nM, respectively (Deane et al., 2004). In contrast, Yamada et al. (2008) reported that Aβ did not show any significant binding to these LRP1 domains immobilized to microtiter plates in a solid phase binding assay.

It is important to note that HSPG also serves as major Aβ binding receptor on the cell surface (Kanekiyo et al., 2011). HSPG mediates the entry of diverse molecules such as exosomes, cell penetrating peptides, polycation-nucleic acid complexes, viruses, lipoproteins, growth factors, and morphogens into cells (Christianson and Belting, 2013). The HSPG endocytosis pathway likely varies depending on the cellular context and type of extracellular ligands. In the case of Aβ, HSPG appears to provide an initial binding site for Aβ on the cell surface, where LRP1 then mediates its endocytosis (**Figure 2**; Kanekiyo et al., 2011) by forming LRP1-HSPG complexes (Wilsie and Orlando, 2003). It is also possible that LRP1 constitutively endocytoses Aβ that is bound to cell surface HSPG. GPI-anchored cellular prion protein (PrP^c^), which is localized in cholesterol-rich lipid raft microdomains of the plasma membrane (Taylor and Hooper, 2006), has also been demonstrated to mediate Aβ oligomer binding on the cell surface (Lauren et al., 2009; Wang et al., 2013). Interestingly, LRP1 interacts with PrP^c^ (Jen et al., 2010) and facilitates its clathrin-mediated endocytosis (Taylor and Hooper, 2007). Thus, LRP1 is required for Aβ oligomer-PrP^c^ interaction and internalization (Rushworth et al., 2013). While LRP1 is mainly localized in non-raft regions of the plasma membrane, it is also known to interact transiently with lipid rafts under specific conditions (Wu and Gonias, 2005). In addition, cell surface HSPG glypican-1 is co-localized with PrP^c^ and recruits it to lipid rafts (Hooper, 2011). Therefore, LRP1 may form a complex with PrP^c^ and HSPG, and regulate Aβ endocytosis in either clathrin- or caveolin-dependent manner (**Figure 2**).

In addition to functioning as an endocytic receptor, LRP1 may control cellular Aβ uptake by modulating signaling pathways. LRP1 is known to regulate Rac1 and RhoA activities in Schwann cell, which influences cell migration and adhesion (Mantuano et al., 2010). In fact, a part of Aβ42 oligomers is likely internalized through a dynamin-dependent and RhoA-mediated endocytic pathway in neuronal cells (Yu et al., 2010). While dynamin regulates both clathrin or caveolin-dependent and -independent pathways, RhoA-mediated endocytosis does not require clathrin or caveolin (Mayor and Pagano, 2007). Although it is not fully understood how LRP1 mediates the activity of these Rho family GTPases in different cell types, LRP1 may also be involved in RhoA-dependent endocytic mechanisms of Aβ (**Figure 2**). In addition, larger size Aβ aggregates are thought be taken up by cells through macropinocytosis or phagocytosis, where actin polymerization is a critical step (Mayor and Pagano, 2007). Since LRP1 is predicted to control cytoskeleton architectures by modifying phosphoinositide 3-kinase (PI3K)/extracellular signal-regulated kinase (ERK) and/or focal adhesion kinase (FAK)/paxillin pathways (Dedieu and Langlois, 2008), LRP1 may also affect macropinocytosis or phagocytosis of Aβ (**Figure 2**).

## SUMMARY AND PERSPECTIVE

Low-density lipoprotein receptor-related protein 1 regulates cellular Aβ uptake and degradation in neurons, astrocytes, and microglia in brain parenchyma, and in vascular smooth muscle cells and pericytes in cerebrovasculature. It also mediates Aβ clearance at the BBB by facilitating Aβ transport from brain to blood (**Figure 1**). LRP1-mediated cellular Aβ uptake likely depends on diverse molecular mechanisms including: (1) endocytosis of Aβ through direct binding; (2) regulation of trafficking for other Aβ receptors such as HSPG and PrP^c^; (3) regulation of RhoA-dependent endocytosis pathway by controlling Rho family GTPase activity; and (4) micropinocytosis/phagocytosis of Aβ by affecting actin polymerization (**Figure 2**). Thus, LRP1 likely mediates cellular Aβ clearance through several endocytic pathways depending on each brain cell type.

Apolipoprotein E is a major ligand for LRP1 and influences AD risk by affecting Aβ aggregation, cellular uptake and degradation. While decreased apoE levels reduce Aβ deposition (Kim et al., 2011; Bien-Ly et al., 2012), the pharmacological approaches to increase lipidated apoE by liver X receptor (LXR) and retinoid X receptor (RXR) agonists facilitate Aβ clearance and restore cognitive function in amyloid model mice (Riddell et al., 2007; Terwel et al., 2011; Vanmierlo et al., 2011; Cramer et al., 2012). While apoE and Aβ can interact with each other, they also share common receptors including LRP1, LDLR, and HSPG on cell surface. ApoE likely competes with Aβ for their receptor binding but can also facilitate cellular Aβ uptake by forming apoE/Aβ complexes depending on their concentrations, apoE isoform involved, lipidation status, Aβ aggregation status and receptor distribution patterns. Dissecting how LRP1 participates in apoE-mediated Aβ clearance will be critical to develop apoE-targeted therapy for AD.

There have been several studies investigating the effects of altered LRP1 expression on Aβ metabolism. It was shown that treatment with a hydroxymethylglutaryl-CoA reductase inhibitor, fluvastatin, increases LRP1 in mouse cerebral vessels, which results in reduced brain Aβ level (Shinohara et al., 2010). Rifampicin and caffeine also upregulate LRP1 levels at the BBB and enhance Aβ elimination from the mouse brain (Qosa et al., 2012). In peripheral tissues, insulin facilitates the hepatic clearance of plasma Aβ by increasing cell surface LRP1 distribution in hepatocytes (Tamaki et al., 2007), which in turn affects brain Aβ clearance. Given that LRP1 can control Aβ elimination from the brain in a variety of cell types, it might be important to define potentially different LRP1-mediated Aβ clearance mechanisms in each cell type to develop novel AD therapeutic methods, which target LRP1 and its ligands.

Taken together, it is clear that LRP1 plays a critical role in cellular Aβ uptake and brain Aβ clearance. It remains to be elucidated how much of LRP1 function depends on interplay with other mechanisms. Future studies are also needed to address how LRP1 in each cell type participates in AD pathogenesis through Aβ-dependent and/or independent pathways using both *in vitro* and *in vivo* models.

## Conflict of Interest Statement

The authors declare that the research was conducted in the absence of any commercial or financial relationships that could be construed as a potential conflict of interest.
